# A novel nonsense *SMC1A* mutation in a patient with intractable epilepsy and cardiac malformation

**DOI:** 10.1038/s41439-019-0053-y

**Published:** 2019-05-13

**Authors:** Yasutsugu Chinen, Sadao Nakamura, Takuya Kaneshi, Mami Nakayashiro, Kumiko Yanagi, Tadashi Kaname, Kenji Naritomi, Koichi Nakanishi

**Affiliations:** 1 Department of Child Health and Welfare (Pediatrics), Graduate School of Medicine, University of the Ryukyus, Nishihara, Japan; 2 Department of Pediatrics, Okinawa Prefectural Nanbu Medical Center Children’s Medical Center, Haebaru, Okinawa Japan; 3 Department of Genome Medicine, National Center for Child Health and Development, Tokyo, Japan; 4 Okinawa Nanbu Habilitation and Medical Center, Naha, Japan

**Keywords:** Disease genetics, Genetic counselling

## Abstract

Cornelia de Lange syndrome (CdLS) is a cohesinopathy caused by genetic variations. We present a female with *SMC1A*-associated CdLS with a novel *SMC1A* truncation mutation (p. Arg499Ter), transposition of the great arteries, and periodic intractable seizures from 40 months of age. A review of the literature revealed that a seizure-free period after birth of at least 15 months is required for these patients to be able to walk, irrespective of the epileptic course.

Cornelia de Lange syndrome (CdLS) [MIM: 122470, 300590, 300882, 610759, and 614701] is a congenital multisystemic disorder with widely varied characteristics ranging from mild (nonclassical phenotype) to severe (classical phenotype) that is caused by genetic variants of structural or regulatory components of the cohesin complex^1,2^. Classical CdLS is caused by mutations in *NIPBL*, while nonclassical CdLS is caused by mutations in *SMC1A*, *SMC3A*, *RAD21*, and *HDAC8*^1^. Missense variants and small in-frame deletions in *SMC1A*, located at Xp11.22, account for ~5% of CdLS cases. A review of the literature revealed 60 cases of *SMC1A*-associated CdLS with a male-to-female ratio of 1:2^3^. A total of nine cases of CdLS with congenital cardiac defects (CHD) have been reviewed^4^, although the incidence of CHD in patients with CdLS is ~30%^5^. *SMC1A*-related CdLS arises from a dominant negative effect in females^2^. Females with *SMC1A* mutations leading to protein truncation are affected by intractable epilepsy, severe developmental retardation, and few craniofacial differences^4,6^. Age at presentation with first epileptic seizures ranges from <1 month to 17 months^6^.

In our case of CdLS with an *SMC1A* truncating mutation, the female patient was the second child born to a healthy, nonconsanguineous couple when her mother and father were 35 and 42 years of age, respectively. There was no family history of CdLS. She was born via emergency cesarean section at 35 weeks gestation due to fetal distress. At birth, her weight was 1636 g (−1.9 SD), length was 43.5 cm (−0.43 SD), and occipitofrontal circumference (OFC) was 30.2 cm (−0.6 SD). She had been hospitalized in the neonatal intensive care unit for 3 months because of failure to thrive and transposition of the great arteries (TGA) type III congenital heart defects with a ventricular septal defect and pulmonary artery stenosis. At 11 months of age, the Blalock–Taussig shunt operation was performed, followed by home oxygen therapy. At 1 year 10 months of age, the Nissen operation was performed for gastroesophageal reflux disease. At 3 years 4 months of age, tonic seizures emerged. Hypoglycemia and high ammonia levels appeared. Hyperammonemia (405 µg/dl; normal range: 36–86 µg/dl) and repetitive hypoglycemia occurred after she suffered from bacterial pneumonia at 4 years 7 months of age. She had been taking six meals a day, two with cornstarch. She was referred to our clinic for detailed examination of metabolic diseases. At 4 years 9 months, she weighed 14.1 kg (−1.3 SD), her height was 100.5 cm (−1.1 SD), and her OFC was 46.8 cm (−2.2 SD). She had a prominent forehead, hypertelorism, thick eyebrows, broad nasal tip, depressed nasal bridge, full cheeks, left cupped ear, right prominent antihelix, high-arched palate, thin upper lip, crowded teeth, slender fingers, left talipes varus, and left second short toe (Fig. 1a-g). The results of all biochemical tests and gas chromatography/mass spectrometry (GC/MS) of urine and tandem mass spectrometry (MS/MS) of dry blood spots were normal. Hypoglycemia was observed during hospitalization, and occasionally episodes caused hypoglycemia irrespective of meal time. Following this, there were no episodes of hypoglycemia for 3 years. A fasting test was conducted at 7 years 3 months of age. Hypoglycemia (37 mg/dl; normal range 70–105 mg/dl) appeared 17 h after the last meal, but there were no findings of coldness, lethargy, hyperammonemia, or metabolic acidosis. Additionally, all urine GC/MS and dry blood spot MS/MS results were normal. Magnetic resonance imaging of the brain revealed normal findings. She had left sensorineural deafness (100 dB). Her developmental milestones were delayed: head control at 6 months of age, rollover at 6 months, sitting unaided at 2 years and 4 months, and walking at 2 years and 5 months. At 15 years 2 months of age, she was unable to speak meaningful words, her height was 133.0 cm (−4.6 SD), and her weight was 27.1 kg (−3.0 SD). Her menstruation started regularly at 14 years 9 months of age. At 13 years of age, six persistent deciduous teeth with no dental caries were extracted.

**Fig. 1:**
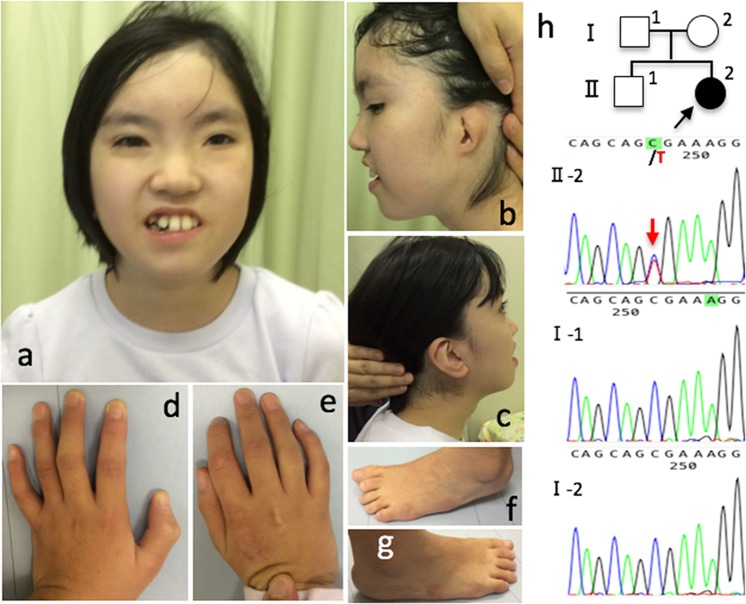
Patient’s appearance at 12 years of age. Face (front, **a**; lateral, **b** and **c**), hand (left, **d**; right **e**), and foot (left, **f**; right, **g**). Sequencing profiles of the *SMC1A* gene of the patient and her parents are shown (**h**)

Three-day-long continuous intractable generalized tonic seizures (GTS) occurred every 2 weeks from 3 years to 4 months of age. At 15 years of age, moderately beneficial antiepileptic drugs (AEDs) were lamotrigine (LMT) and levetiracetam (LEV). AEDs tried without success were clobazam (CLB), potassium bromide (KBr), and carbamazepine (CBZ). She had a Jatene operation for TGA type III at 5 years 2 months of age and corrective surgery for the left talipes varus at 6 years 10 months. Chromosomal analysis showed a normal karyotype. We searched the original computerized database for possible malformation syndromes: UR-DBMS (University of the Ryukyus-Database for Malformation Syndromes: http://becomerich.lab.u-ryukyu.ac.jp) edited by Naritomi^7^. Suggested candidates matching over 12 signs were 4pter-p13 trisomy and Xpter-p21 monosomy, 13q14-qter trisomy and 5pter-p13 monosomy, CDLS1 (MIM 122470), CDLS3 (MIM 610759), Noonan syndrome 1 (NS1) (MIM 163950), 1q21.1 deletion syndrome, 12p trisomy, and 16p11.2 deletion syndrome. Whole-exome sequencing was performed using the SureSelect Human All Exon V6 Kit (Agilent Technologies, Santa Clara, CA) and HiSeq2500 (Illumina, San Diego, CA). To identify disease-causing mutations, we excluded all known variants found in the 1000 Genomes database (http://www.internationalgenome.org/), Japanese Genomes database^8^, dbSNP (http://www.ncbi.nlm.gov/SNP), the genome Aggregation Database (gnomAD; http://gnomad.broadinstitute.org/), and the Human Genetic Variation Database (HGVD; http://www.genome.med.kyoto-u.ac.jp/SnpDB/). Heterozygous *SMC1A* (NM_006306) mutations cause CdLS, the symptoms of which fit with those of our patient. We identified a heterozygous single nucleotide variation (c. C1495T) in *SMC1A* exon 9 that results in a nonsense mutation (p. Arg499Ter) and a truncated protein. The p. Arg499Ter variant was not detected in her parents, suggesting that the variant was de novo. This was confirmed by Sanger sequencing (Fig. 1h). This is the first description of the p. Arg499Ter variant. Mutation Taster (http://www.mutationtaster.org) predictions indicate that this is a disease-causing variant. This study was performed in accordance with the standards of the Ethics Committee of the Ryukyus Graduate School of Medicine (Okinawa, Japan). Informed consent was obtained from her parents by Dr. Yasutsugu Chinen.

Our patient with *SMC1A*-associated CdLS had a nonclassical CdLS type. This was determined using the clinical CdLS scoring system, which consists of 9 points, including three cardinal and three suggestive features^1,6^. Her first seizures, which occurred at 40 months of age, were the latest onset recorded. Seizure onset occurred much earlier in the 15 previously reported patients with protein truncating mutations in *SMC1A*^6^. When these cases, including the one presented here, were arranged in order of age of first seizures, we observed that only one (9.1%) of the 11 patients who had their first seizure at less than 9 months of age could walk. In contrast, all of the patients (100%) who had their first seizure after 15 months could walk. This observation was independent of the type of epilepsy and the truncated amino acid position in SMC1A (Table 1, modified from Symonds et al. 2017)^6,9–11^. Although the number of cases is small, this observation suggests a correlation between age of epilepsy onset and independent walking. If the first seizures develop before the patient could walk, walking without assistance may be difficult. Only one patient, case 9, developed seizures in the 5 months after birth and was able to walk at 30 months of age. This patient was seizure-free for 1 year after commencement of levetiracetam treatment, but the seizures recurred. In case 4, seizures started from 5–6 weeks of age and stopped occurring at 5 years of age after commencement of phenobarbitone treatment and adoption of a ketogenic diet. In case 8, seizures started from 5 months of age and stopped occurring at 7 years of age after commencement of gabapentin treatment and adoption of a ketogenic diet. Taken together, these cases indicate that at least the first 15 months after birth needs to be seizure-free for these patients to walk, irrespective of the epileptic course.

**Table 1:** Characteristics in order of age of first seizures in our patient and previously reported cases

Feature	Case 1	Case 2	Case 3	Case 4	Case 5	Case 6	Case 7	Case 8	Case 9	Case 10	Case 11	Case 12	Case 13	Case 14	Case 15	Case 16
Age at reported (years)	Died aged 11 M	7	4	6	Died aged 9Y2M	4	3	8	5	10	46	6	3	14	14	15
Birth OFC Z-score/ most recent	−1.7/Unkonwn	−3.9/−2.5	Unknown/−2.0	−1.5/−4.5	−1.3/−6.3	Unknown/−2.0	−1.6/−0.8	−2.0/−2.5	Unknown/−3.0	−1.2/−2.0	Unknwn/−2.5	−0.8/−3.5	−1.0/0.0	Unknown/−1.7	Unknown/−2.0	−0.6/−2.2
Most recent height Z-score	Unknown	−2	−2.5	−2.6	−5	Unknown	0.006	−2.6	−3.2	−4.5	−2.5	−2.3	−0.05	−2	−3.7	−4.6
Developmental impairment	Unknown	Unknown	Moderate-severe	Severe	Unknown	Unknown?	Severe	Severe	Moderate-severe	Severe	Unknown	Moderate-severe	Unknown	Unknown	Moderate-severe	Severe
Gross motor development	None	Unable to sit	Can take a couple of steps with support	Unable to sit without support	Unable to sit	Non ambulant	Unable to sit without support	No independent mobility	Walking from 30 months	Unable to sit without support	Never crawled or walked	Run with unsteady gait	Walked at 12 months	Walked at 2 year; suddenly stopped walking at 5 years	Walking from 2.5 years. Unsteady on feet, aged 7	Walking from 2.4 years
Speech	None	None	None. Coos, laughs, cries appropriately	None	None	None	None	None	Lost speech aged 3 years following SE	None	None	None. Smiles and makes hand gestures	None. Coos, interacts	None	None	None
Age at first seizure	<1 month	<1 month	4 weeks	5–6 weeks	2 months	4 months	4 months	5 months	5 months	6 months	9 months	15 months	17 months	2 years	28 months	40 months
Seizure types	Unknown	Focal with eyelid myoclonia, Focal, spasms	Bilateral clonic, GTCS, hemiclonic	GTCS, Focal	GTCS, myoclonic, CSE	Generalized tonic, Tonic, focal → bilateral clonic, CSE	Generalized tonic, FS, CSE, focal, myoclonic, spasms, tonic, atypical absence	Focal → bilateral clonic, Focal, generalized tonic	Focal → bilateral tonic focal → bilateral clonic	GTCS, myocloic, atypical absence, tonic, spasms, NCSE, reflex sensory	GTCS	Cluster of GTCS, GTCS, hemiclonic, drop attacks, atypical absence	Focal.atypical absence, GTCS	GTCS	FS, GTCS	Generalized tonic
Seizure clusters	−	−	+	−	−	+	+	+	+	+	+	+	+	+	+	+
Seizure freedom	−	−	−	+(5 years of age)	−	−	−	+(7 years of age)	+(for 1 year then recurred)	−	−	−	+	−	−	−
SMC1A:Amino acid change	c.2477delA Frameshift	p.Thr638Valfs*48	p.Arg171Ter	p.Arg1049Ter	p.Gln1039Ter	p.Ser951Argfs*12	p.Glu183Glu	p.Glu733Ter	p.Arg975Ter	p.Asp1109AlafsTer102	p.Asn788Lysfs*10	p.Gln531Ter	p.Ile1185Glyfs*23	p.Leu808Argfs*6	c.2477delA Frameshift	p.Arg499Ter
Reference	6	9	6	6	6	10	6	6	6	6	11	6	10	11	6	This report

*GTCS* generalized tonic-clonic sezure, *CSE* convulsive status epilepticus, *FS* febrile seizure, *NCSE* nonconvulsive status epilepticus

CHD with *SMC1A*-associated CdLS has been previously reported in 13 cases, including ours, yet this is the first report including a TGA diagnosis (Supplementary Table 1)^2–4,6,12–18^. In patients with CHD, the rate of substitution or mutation at an arginine position is 42% (5/12), and in patients without CHD, the rate is 50% (11/22). The post-translational modification of proteins by arginine methylation is functionally important in the SMC1A protein^19^. Dysregulation of cohesin by the SMC1A protein may cause CHD in a zebrafish cohesinopathy model^20^. The same variations at arginine positions, including p. Val58-Arg62del, p. Arg196, p. Arg496, and p. Arg693, revealed discordant heart defects. However, there may be no relationship between SMC1A arginine substitution and CHD, and we were unable to show whether such SMC1A variations affect arginine methylation.

Our patient’s episodes of hypoglycemia showed no remarkable causal disease and were considered clinical symptoms caused by dumping syndrome. However, we could not regularly observe reproducible events. The fasting test at 7 years 3 months of age revealed a normal reaction without hypoglycemic episodes. Infancy is considered to be a time period of low glycogen storage corresponding to age. If hypoglycemia occurs during this period, further careful investigation and dietary adjustments might be necessary.

## Supplementary information

Supplementary Table 1

## Data Availability

The relevant data from this Data Report are hosted at the Human Genome Variation Database at 10.6084/m9.figshare.hgv.2570.
